# White matter lesions in migraine: a single-center retrospective study of prevalence and risk factors

**DOI:** 10.3389/fneur.2026.1862788

**Published:** 2026-07-29

**Authors:** Nabil Akkawi, Elkhansa Hassabo Mohamed, Marwa Suliman, Marim Bakhiet, Tamer Mostafa

**Affiliations:** Department of Neurology, HMS Mirdif Hospital, Dubai, United Arab Emirates

**Keywords:** magnetic resonance imaging, migraine, MRI, white matter lesions, WML

## Abstract

**Introduction:**

Migraine is ranked as the third most common and second most disabling neurological disorder worldwide. Neuroimaging studies demonstrate higher white matter lesion (WML) prevalence in migraineurs, with a meta-analysis reporting a 44% pooled prevalence. In the UAE, migraine affects 18.2% of healthcare professionals, but WML prevalence remains unstudied. This study investigates WML prevalence and clinical correlates in UAE migraine patients.

**Methods:**

This retrospective study included 313 migraine patients aged 10–50 years who underwent brain MRI at HMS Mirdif Hospital (2022–2025). MRI scans (3T, T2/FLAIR) were assessed by three trained doctors supervised by a radiologist. Demographic, clinical, and MRI data were collected from electronic records. Non-parametric tests and binary logistic regression were used (*p* < 0.05).

**Results:**

A total of 313 migraine patients (median age 32 years, 73.8% female) were included. White matter lesions were identified in 35.5% (*n* = 111), with a median of 4 lesions (IQR: 2–10). Lesions were predominantly frontal (86.5%), bilateral (70.3%), and cortical–subcortical (94.6%). Age was the only significant predictor of WML presence (OR = 1.038, *p* = 0.003), but did not correlate with lesion number among affected patients.

**Conclusion:**

This study found white matter lesions in 35.5% of migraine patients, predominantly affecting frontal and cortical–subcortical regions. Age was the strongest predictor of lesion presence but did not correlate with lesion number. Why WMLs develop in some patients but not others remain unclear. The retrospective single-center design, potential selection bias, limited generalizability, and lack of an age- and sex-matched control group warrant cautious interpretation and underscore the need for prospective studies.

## Introduction

Migraine is a complex neurological condition characterized by recurrent headache attacks, autonomic and sensory disturbances, and, in approximately one-third of cases, reversible localized neurological manifestations known as aura (1). The World Health Organization ranks migraine as the third most common and second most disabling neurological disorder worldwide. According to the Global Health Estimates 2021, migraine was the third leading neurological cause of disability-adjusted life years (DALYs) worldwide, following stroke and neonatal encephalopathy. Not all neurological disease burden is attributable to population aging, highlighting the importance of quantifying the overall health loss associated with nervous system disorders across the lifespan (2, 3).

With the increasing utilization of magnetic resonance imaging (MRI) in clinical practice and research, multiple studies have identified a higher prevalence of white matter lesions (WMLs)—also referred to as white matter hyperintensities—in individuals with migraine compared to the general population (4, 5). These lesions appear as hyperintense areas on T2-weighted and fluid-attenuated inversion recovery (FLAIR) sequences and are predominantly located in the deep and periventricular white matter regions (4, 6). Notably, WMLs have been documented in relatively young migraine patients who lack traditional vascular risk factors, suggesting a migraine-specific mechanism (4, 6).

A recent meta-analysis of observational studies involving over 3,500 migraineurs reported a pooled WML prevalence of approximately 44%, with comparable rates observed in patients with aura (45%) and those without aura (38%). The frontal lobes and subcortical white matter represent the most frequently affected regions, suggesting a characteristic spatial distribution pattern in migraine (7). Furthermore, individuals with migraine demonstrate a significantly higher burden of WMLs than age-matched non-migraine controls, exhibiting more than four times the likelihood of having such lesions (7). Several factors have been consistently linked to an increased burden of these lesions, including female sex, higher frequency of migraine attacks, and longer disease duration (4, 7). However, data across studies remain heterogeneous, and the strength of these associations varies considerably according to study design, imaging protocols, and population characteristics (5).

Several pathophysiological mechanisms have been proposed to explain the association between migraine and the development of WMLs. These include recurrent cortical spreading depolarization with transient cerebral hypoperfusion, microvascular ischemic injury, endothelial dysfunction, impaired cerebrovascular autoregulation, and neuroinflammation (8, 9). Reduced cerebral perfusion pressure and sluggish cerebral blood flow have been observed during and after migraine attacks (10–12). During migraine attacks, decreased cerebral perfusion may impair the clearance of embolic particles, while occlusive thrombi may further compromise blood flow (13). Supporting this hypothesis, impaired cerebrovascular reactivity/autoregulation has been reported in migraine patients with WMLs, including increased levels of asymmetric dimethylarginine, an endogenous inhibitor of nitric oxide synthase with vasoconstrictive effects, compared with migraine patients without WMLs and healthy controls (14). Several observations support episodic ischemia as a possible contributor to WML formation in migraineurs. Migraine has been associated with endothelial dysfunction (15, 16), hypercoagulability (17), atrial fibrillation (18), and an increased prevalence of patent foramen ovale, which may facilitate cerebral microembolism (19). In addition, migraineurs may exhibit increased interictal vasoconstrictive tendency (20). Neuroinflammation may also contribute, as migraineurs show evidence of a proinflammatory baseline state, with elevated peripheral levels of IL-1β, IL-6, and tumor necrosis factor-α. During migraine attacks, serum levels of C-reactive protein, von Willebrand factor, IL-1β, IL-6, IL-8, and tumor necrosis factor-α may increase further (21). Despite these proposed mechanisms, definitive evidence establishing a causal link between migraine and WML development remains lacking (5).

The clinical implications of white matter abnormalities in migraine patients remain incompletely understood and represent an area of active investigation. While many lesions appear to be clinically silent and are often discovered incidentally on neuroimaging, growing concerns have been raised regarding their potential association with cognitive dysfunction, increased risk of ischemic stroke, and long-term neurological sequelae (4, 22).

Migraine is a major neurological health concern in the UAE. According to epidemiological data, migraine is relatively common in the UAE population, with a prevalence of approximately 18.2% among healthcare professionals, demonstrating its significant impact on productivity and quality of life, particularly among females and those exposed to occupational stressors and sleep disturbances (23). Regional studies from the Middle East show a wide prevalence range of migraine, from 2.6 to 32%, reflecting methodological and demographic diversity but highlighting the disease’s significant burden in Gulf countries, including the UAE (24).

Despite the growing body of international evidence linking migraine with white matter hyperintensities, significant knowledge gaps persist. The prevalence and characteristics of WMLs in migraine patients have not been systematically examined in the UAE population, where genetic, environmental, and lifestyle factors may influence both migraine expression and its neuroimaging correlates. Given the high prevalence of migraine in the UAE and the potential clinical implications of WMLs, region-specific data are urgently needed to guide local clinical practice and contribute to the global evidence base.

The purpose of this study is to investigate the prevalence and neuroimaging characteristics of WML in migraine patients in the UAE, and to examine their association with clinical and demographic parameters.

## Methods

### Study design and settings

This was a single-center retrospective cross-sectional study conducted at HMS Mirdif Hospital, a tertiary care neurological center, between January 2022 and July 2025.

### Study aim

The study aimed to characterize WMLs in migraine patients and identify associated clinical and demographic factors.

### Study population

Patients diagnosed with migraine according to the International Classification of Headache Disorders, 3rd edition (ICHD-3) criteria who underwent MRI were included. All patients included in the study underwent brain MRI for a clinical indication as part of routine neurological evaluation. The primary purpose of MRI was to exclude alternative diagnoses that may mimic migraine or present with headache, including space-occupying lesions, structural abnormalities, vascular lesions, demyelinating disorders, or other secondary causes of headache.

### Inclusion criteria

Patients (age ≥10 years) diagnosed with migraine who underwent an MRI with clinical indication. Patients with DMII, hypertension and obesity were included.

### Exclusion criteria

Patients with history of Stroke, Transient Ischemic Attack (TIA), or Cerebrovascular disease, Neurodegenerative disease and Multiple Sclerosis (MS).

Additionally, patients older than 50 years were excluded to avoid including white matter hyperintensities attributable to chronic cerebral small vessel disease.

### Sample size determination

Sample size was determined using convenience sampling of all eligible patients during the study period. A total of 313 migraine patients who underwent brain MRI during the study period met inclusion criteria.

### Study instruments

Neuroimaging Protocol:

Brain MRI was performed using a 3.0 Tesla Siemens system. WMLs were assessed using FLAIR sequences.

### Data collection

Data were collected through review of electronic medical records. Clinical variables included: Age, Gender, Nationality, Migraine type (episodic/chronic), Aura presence, Monthly Migraine Days (MMD), comorbidities (hypertension, diabetes mellitus), Body Mass Index (BMI).

MRI examinations were initially interpreted by a neuroradiologist as part of routine clinical practice. For the purposes of this study, imaging data were independently reviewed and extracted by trained physicians under the supervision of the neuroradiologist.

### Data analysis

Statistical analysis was performed by E. Mohamed, the corresponding author, using IBM SPSS Statistics version 23. Artificial intelligence was not used in the data analysis; it was employed solely for language editing and to enhance the readability and clarity of the Results section (DeepSeek). Continuous variables were assessed for normality using the Shapiro–Wilk test and visual inspection of histograms. All continuous variables—Age, Monthly Migraine Days (MMD), and WML numbers—were not normally distributed (*p* < 0.001). Consequently, non-parametric tests were employed. Data are presented as median with interquartile range (IQR) unless otherwise specified.

Descriptive statistics were reported as frequencies and percentages for categorical variables. Group comparisons for continuous variables were conducted using the Mann–Whitney *U* test for two independent groups and the Kruskal–Wallis test for three or more groups. Chi-square or Fisher’s exact tests were used for categorical variables. Associations between continuous non-normally distributed variables were assessed using Spearman’s rank-order correlation (*ρ*). Binary logistic regression was used to identify predictors of WML presence (yes/no), with results expressed as odds ratios (OR) and 95% confidence intervals (CI). A two-sided *p*-value <0.05 was considered statistically significant.

### Ethical considerations

The study protocol was approved by the Institutional Review Board of HMS Mirdif Hospital. The research adhered to the principles of the Declaration of Helsinki. Patient confidentiality was maintained through anonymized data coding, with access restricted to authorized investigators.

### Quality assurance

White matter lesions were defined as hyperintense foci on T2-weighted and FLAIR MRI sequences. To ensure data quality and consistency of lesion identification, 25% of MRI studies were randomly selected and independently reviewed by a consultant neurologist who is blinded to the initial research review. Any discrepancies were resolved by consensus with the supervising neuroradiologist. The study focused on the presence and anatomical distribution of white matter lesions; therefore, a formal lesion severity grading scale was not applied.

### Variable definitions

- Chronic migraine: ≥15 headache days/month for >3 months.
- Episodic migraine: <15 headache days/month for >3 months.
- White matter lesions: hyperintense areas on T2-FLAIR sequences.
- WML presence: ≥1 hyperintense lesions within white matter.
- Periventricular WMLs: lesions within 1 cm of lateral ventricles.
- Cortical WMLs: lesions within the cortex.
- Subcortical WMLs: lesions beneath the cortex.
- Bilateral involvement: lesions present in both cerebral hemispheres.

## Results

### Demographic and clinical characteristics

This study included 313 migraine patients with a median age of 32 years (IQR: 24–41 years). The cohort demonstrated a marked female predominance (73.8%, *n* = 231), and most patients were of local nationality (75.7%, *n* = 237). Regarding migraine characteristics, patients reported a median of 12 monthly migraine days (IQR: 4–30), with episodic migraine being more common (54.6%, *n* = 171) than chronic migraine (45.4%, *n* = 142). Only 14.7% (*n* = 46) of patients experienced aura.

The participants had relatively few traditional vascular risk factors. Diabetes mellitus was uncommon (2.9%, *n* = 9), as was hypertension (5.8%, *n* = 18). BMI distribution revealed 46.0% (*n* = 144) of patients in the normal range (<25 kg/m^2^), 34.8% (*n* = 109) overweight (25–30 kg/m^2^), and 19.2% (*n* = 60) obese (>30 kg/m^2^), As shown in Table 1.

**Table 1:** Demographic and clinical characteristics among patients with migraine (*n* = 313).

Variables		Median (IQR)	Count	Percentage %
Age		32 (24–41)		
Gender	Male		82	26.2%
	Female		231	73.8%
Nationality	Local		237	75.7%
	Non local		76	24.3%
MMD^a^		12 (4–30)		
Migraine type	Episodic migraine		171	54.6%
	Chronic migraine		142	45.4%
Aura	Yes		46	14.7%
	No		267	85.3%
DM^b^	Yes		9	2.9%
	No		304	97.1%
HTN^c^	Yes		18	5.8%
	No		295	94.2%
BMI^d^	Less than 25		144	46.0%
	25–30		109	34.8%
	More than 30		60	19.2%
WML^e^ presence	Yes		111	35.5%
	No		202	64.5%
WML numbers		4 (2–10)		

a Monthly migraine days.

b Diabetes mellitus.

c Hypertension.

d Body Mass Index.

e White matter lesions.

### WMLs percentage

WMLs were identified in 35.5% (*n* = 111) of patients. Among those with WMLs, the mean lesion count was 7.4 (SD = 9.1), the median was 4 lesions (IQR: 2–10, range: 1–48). The distribution remained highly right-skewed (skewness = 2.5, kurtosis = 7.1).

### WML anatomical distribution

**Figure 1:**
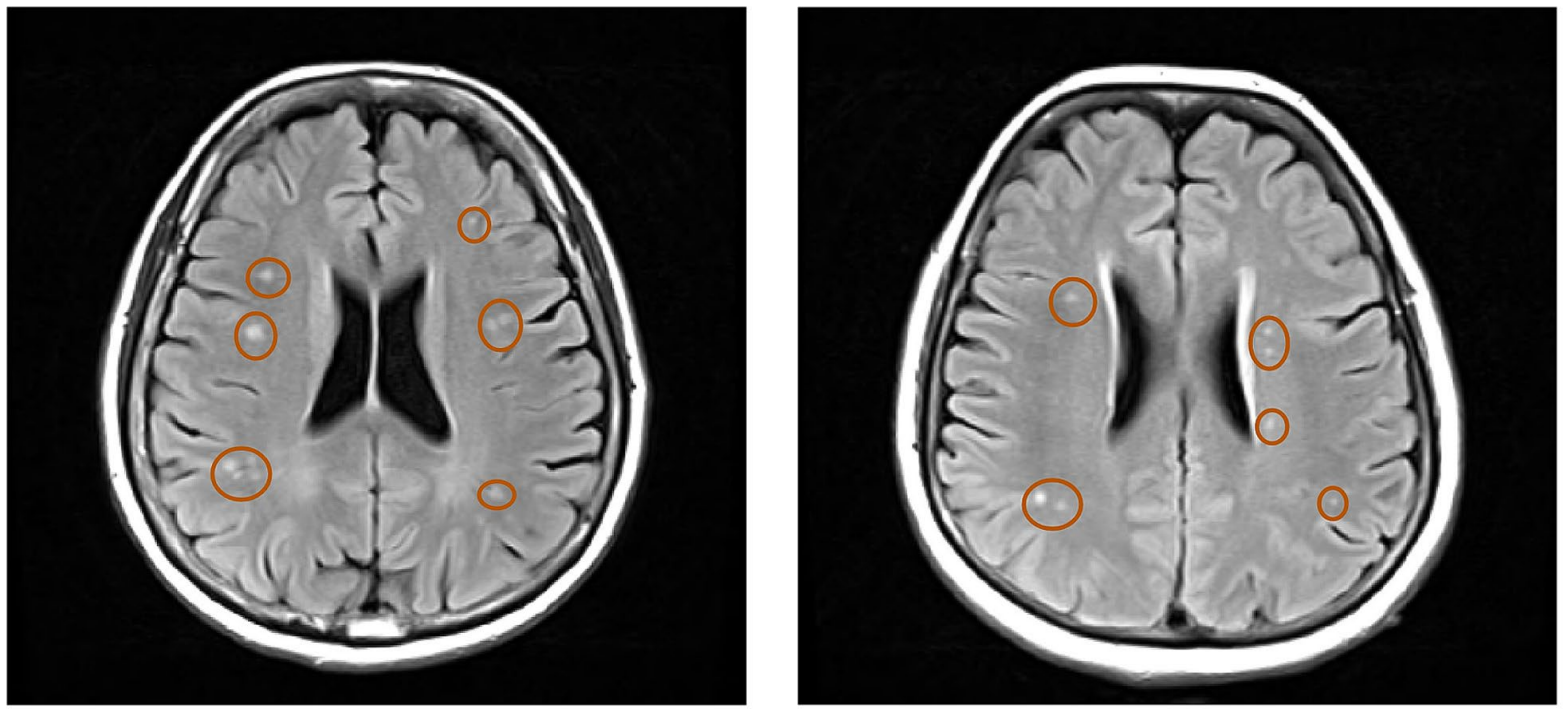
Axial T2-FLAIR MRI from two different patients with migraine. Image 1 (left) shows subcortical white matter hyperintensities (WMH) in a frontoparietal distribution. Image 2 (right) demonstrates WMH in a periventricular in addition to in a frontoparietal distribution.

Frontal lobe involvement was predominant, with isolated frontal lesions in 31.5% (*n* = 35) and combined frontal–parietal lesions in 55.0% (*n* = 61) of the cohort. More extensive multi-lobar involvement (three or more lobes) was infrequent (6.3%, *n* = 7). As demonstrated in Figure 1, Table 2.

**Table 2:** WML anatomical distribution among patients with migraine (*n* = 313).

Variables		Count	Column *N* %
WML^a^ lobe	Frontal	35	31.5%
	Parietal	5	4.5%
	Temporal	1	0.9%
	Occipital	1	0.9%
	Frontal and parietal	61	55.0%
	Frontal and temporal	1	0.9%
	Frontal, parietal and temporal	6	5.4%
	Frontal, parietal, temporal and occipital	1	0.9%
WML cortical–subcortical	Yes	105	94.6%
	No	6	5.4%
WML periventricular	Yes	22	19.8%
	No	89	80.2%
WML infratentorial	Yes	0	0.0%
	No	111	100.0%
WML basal ganglion	Yes	2	1.8%
	No	109	98.2%
WML laterality	Unilateral	33	29.7%
	Bilateral	78	70.3%

a White matter lesions.

Regarding spatial distribution, bilateral WMLs (70.3% of cohort, *n* = 78) were more prevalent than unilateral lesions (29.7%, *n* = 33). cortical–subcortical lesions represented the most common location (94.6% of cohort, *n* = 105), followed by periventricular lesions (19.8%, *n* = 22). Infratentorial lesions were absent (0.0%, *n* = 0), and basal ganglion lesions were exceptionally rare, occurring in only 1.8% (*n* = 2) of patients. As summarized in Table 2.

### Factors associated with WML presence in migraine patients

Age was a significant factor associated with WML presence. Patients with WMLs were significantly older than those without lesions (Mean Rank: 176.47 vs. 146.30; *U* = 9049.5, *p* = 0.005). Other factors, including Monthly Migraine Days, Gender, Nationality, Aura presence, and Migraine type, were not significantly associated with WML presence on chi-square test (all *p* > 0.05), as shown in Table 3.

**Table 3:** Demographic and clinical characteristics by WML status among patients with migraine (*n* = 313).

Variables		WML^a^ presence						*p*-value
		Yes			No			
		Median	Count	Column *N* %	Median	Count	Column *N* %	
Age		34			31			0.005*
Gender	Male		28	25.2%		54	26.7%	0.772
	Female		83	74.8%		148	73.3%	
Nationality	Local		86	77.5%		151	74.8%	0.591
	Non local		25	22.5%		51	25.2%	
MMD^b^		12			14			0.513
Migraine type	Episodic migraine		63	56.8%		108	53.5%	0.576
	Chronic migraine		48	43.2%		94	46.5%	
Aura	Yes		18	16.2%		28	13.9%	0.574
	No		93	83.8%		174	86.1%	
DM^c^	Yes		3	2.7%		6	3.0%	1.000
	No		108	97.3%		196	97.0%	
HTN^d^	Yes		8	7.2%		10	5.0%	0.412
	No		103	92.8%		192	95.0%	
BMI^e^	Less than 25		49	44.1%		95	47.0%	0.709
	25–30		38	34.2%		71	35.1%	
	More than 30		24	21.6%		36	17.8%	

a White matter lesions.

b Monthly migraine days.

c Diabetes mellitus.

d Hypertension.

e Body Mass Index.

Binary logistic regression was performed to identify predictors of WML presence, including the following covariates: Age, Monthly Migraine Days (MMD), Gender, Aura, diabetes mellitus (DM), and Migraine type. Age emerged as the sole significant predictor (OR = 1.038, 95% CI: 1.012–1.064, *p* = 0.003), indicating a 3.8% increase in WML odds per year of age. All other covariates were non-significant as shown in Table 4.

**Table 4:** Binary logistic regression results for predictors of white matter lesion (WML) presence.

Variables in the equation		*B*	SE	Wald	df	*p*-value	OR	95% CI	
								Lower	Upper
Step 1^a^	Age	0.037	0.013	8.584	1	0.003	1.038	1.012	1.064
	MMD	0.006	0.018	0.130	1	0.718	1.007	0.972	1.043
	Gender (1)	−0.056	0.278	0.041	1	0.840	0.945	0.548	1.631
	Aura (1)	−0.207	0.340	0.369	1	0.543	0.813	0.418	1.584
	DM (1)	0.087	0.731	0.014	1	0.905	1.091	0.260	4.575
	Migraine type (1)	0.215	0.392	0.300	1	0.584	1.239	0.575	2.673
	Constant	−1.918	1.031	3.461	1	0.063	0.147		

a Variable(s) entered on step 1: Age, MMD, Gender, Aura, DM, Migraine type.

### Factors associated with WML burden (numbers) in migraine patients

Although age was significantly associated with WML presence, it did not correlate with the numbers of lesions in patients who already had WMLs (Spearman’s rho = 0.160, *p* = 0.093). Similarly, monthly migraine days (MMD) showed no correlation with lesion burden (Spearman’s rho = 0.119, *p* = 0.212).

WML burden in migraine patients varies significantly by anatomical distribution. Mann–Whitney *U* tests revealed that bilateral lesions were associated with substantially higher lesion counts than unilateral lesions (Mean Rank: 70.76 vs. 21.11, *p* < 0.001). Periventricular location was associated with higher lesion burden, with affected patients exhibiting higher lesion counts than those without (Mean Rank: 71.48 vs. 52.17, *p* = 0.011). Cortical–subcortical involvement, present in nearly all WML-positive patients (105/111, 94.6%), was also associated with moderately higher lesion burden (Mean Rank: 57.51 vs. 29.50, *p* = 0.037). Finally, a Kruskal–Wallis test showed that lobar distribution was a significant determinant of burden (*χ*^2^ = 36.77, df = 7, *p* < 0.001), with four-lobe involvement (Frontal, Parietal, Temporal and Occipital) having the highest mean rank (90.00). No significant associations were found with demographic factors (Gender, Nationality), clinical features (Aura, Migraine type, comorbidities), or BMI.

## Discussion

This is the first study of its kind conducted in the United Arab Emirates with a relatively large sample size, providing valuable local insights and enabling comparison with previous regional and international studies. In this study of 313 migraine patients, WMLs were found in 35.5% of subjects. This prevalence is substantially higher than that reported in non-migraine control populations, in whom WMLs are typically uncommon, generally ranging from 5 to 15%, highlighting the increased burden among migraine patients (7, 25, 26).

Our finding of 35.5% is somewhat lower than the 44% pooled prevalence reported in a recent meta-analysis of white matter hyperintensities in migraine (7). This difference is best explained by our study design. We excluded patients over 50 years of age to avoid confounding by age-related atherosclerotic white matter changes, allowing us to better isolate migraine-specific pathology. Many studies included in the meta-analysis did not apply such age restrictions and enrolled older participants, which would be expected to yield higher prevalence figures. Nevertheless, we still observed WMLs in more than one-third of our relatively young cohort. Both diabetes mellitus and hypertension are established risk factors for white matter lesions. However, these patients were intentionally included in the study to assess whether these comorbidities contributed to the presence of white matter lesions within our migraine cohort. To account for their potential confounding effect, diabetes and hypertension were included as covariates in the statistical analysis, and appropriate adjustments were performed. This approach allowed us to evaluate the association between migraine and white matter lesions while controlling for the influence of these vascular risk factors. Excluding these patients would have reduced the representativeness of the study population and limited our ability to assess the contribution of these clinically relevant comorbidities.

Our cohort was not derived from a random population sample but rather from patients with migraine who underwent brain MRI as part of their clinical evaluation. Therefore, the reported prevalence reflects the proportion of white matter lesions within this MRI-referred migraine cohort and may not be representative of the general migraine population.

Age emerged as a significant predictor of WML presence. Patients with lesions were older than those without, consistent with meta-analytic evidence identifying age as a dominant risk factor (7). Importantly, this relationship held despite our restricted age range, suggesting that even modest age differences within younger populations influence lesion development in the migraine brain. However, among patients who already had WMLs, age did not correlate with the number of lesions, indicating that other factors may play a greater role in determining the extent of white matter involvement once the initial threshold for lesion formation has been crossed. No significant associations were observed between WML presence and migraine subtype (episodic vs. chronic) or aura status. This is consistent with the literature showing that while aura or chronicity may contribute to WMH risk, these relationships often diminish after adjusting for age and vascular factors (25).

The anatomical distribution of lesions in our patients aligns closely with previous studies. Lesions were predominantly frontal and cortical–subcortical, with bilateral involvement more common than unilateral (26, 27). Infratentorial and basal ganglia lesions were rare, consistent with the understanding that migraine-associated WMLs typically spare these regions (5, 27). This pattern helps distinguish migraine-related findings from those seen in small-vessel ischemic disease or demyelinating conditions, and carries potential clinical relevance given the role of frontal-subcortical circuits in executive function. Furthermore, the predominant frontal and subcortical distribution of lesions aligns with regions known to be particularly sensitive to microvascular dysfunction, endothelial dysregulation, and altered cerebral perfusion (27). The absence of involvement of deep gray matter structures or infratentorial regions further argues against embolic or inflammatory mechanisms as primary drivers of lesion development (5). The mechanisms underlying this characteristic lesion distribution remain uncertain. Previous studies have proposed several hypotheses, including microvascular dysfunction, endothelial dysregulation, impaired cerebrovascular reactivity, and recurrent alterations in cerebral perfusion associated with migraine attacks, which may contribute to the development of white matter lesions. However, these mechanisms remain speculative, and our findings do not establish a causal relationship between migraine and any specific pathophysiological process (9, 26, 28, 29).

Although several previous studies have reported a higher prevalence of white matter lesions (WMLs) in patients with migraine with aura (25, 29), we did not observe a significant difference between migraine with aura and migraine without aura in our cohort. Several factors may explain this finding. First, the relatively small number of patients with migraine with aura may have limited the statistical power to detect a difference between the groups. Second, our study population was relatively young, and the overall burden of WMLs was low, which may have reduced the ability to identify aura-related differences. Third, migraine characteristics such as disease duration, attack frequency, and migraine severity may have a greater influence on WML development than aura status alone. Fourth, differences in MRI acquisition protocols, magnetic field strength, lesion definitions, and image interpretation across studies may contribute to discrepancies in reported findings. In addition, more recent studies and meta-analyses have shown inconsistent results regarding the association between aura and WMLs, suggesting that aura may not be an independent predictor of WMLs after adjustment for demographic and vascular risk factors. Therefore, the absence of a significant difference in our cohort does not necessarily contradict the existing literature and may reflect differences in study design, population characteristics, and statistical power (30, 31).

Several limitations warrant consideration. We acknowledge that the retrospective single-center design and inclusion of patients who underwent MRI as part of their clinical workup may introduce selection bias, as these patients may not be fully representative of the general migraine population. Patients referred for MRI are often those with atypical headache features, refractory symptoms, new-onset headaches, abnormal neurological findings, patient anxiety, or physician concern regarding secondary causes. Consequently, the prevalence of white matter lesions observed in our cohort may not be directly generalizable to all individuals with migraine in the community. Nevertheless, the study reflects real-world clinical practice in a tertiary neurology setting where MRI is commonly performed to exclude secondary headache disorders and evaluate patients presenting with migraine.

The cross-sectional design prevents causal inference regarding the temporal relationship between migraine and WML development. The single-center setting may limit generalizability to the broader UAE population or to neighboring Gulf countries with different demographic characteristics. As this study did not include an age- and sex-matched non-migraine control group, comparisons with the prevalence of white matter lesions reported in the general population should be interpreted with caution. Any such comparisons are based on published historical control data rather than direct observations within the present study. Therefore we cannot definitively quantify the excess burden attributable to migraine itself, though comparison with published control data suggests a substantial migraine-attributable component. Longitudinal studies are needed to determine whether WMLs progress over time, whether they carry functional consequences for cognition, and whether migraine preventive therapies might modify their development.

## Conclusion

This study demonstrates that WMLs are frequently observed in patients with migraine, with a prevalence of 35.5%. Lesions predominantly affect frontal and cortical–subcortical regions, and their overall prevalence increases significantly with age. Age emerged as the strongest demographic predictor of WML presence. However, among patients who already had WML, age did not correlate with the number of lesions. A key unresolved question remains why WMLs develop in some migraine patients and not others. While providing valuable insights from the United Arab Emirates, this study’s findings are limited by the absence of an age-matched non-migraine control group. Future studies incorporating such controls are necessary to clarify the independent contribution of migraine to WML development.

## Data Availability

The raw data supporting the conclusions of this article will be made available by the authors, without undue reservation.
